# Metabolism-disrupting compounds and metabolic health during the menopausal transition: a narrative review

**DOI:** 10.1038/s41366-026-02125-z

**Published:** 2026-06-15

**Authors:** Heather M. Guetterman, Jodi A. Flaws, Rita S. Strakovsky

**Affiliations:** 1 Department of Food Science and Human Nutrition, Michigan State University, East Lansing, MI USA; 2 Institute for Integrative Toxicology, Michigan State University, East Lansing, MI USA; 3 Department of Comparative Biosciences, University of Illinois, Urbana-Champaign, IL USA

**Keywords:** Epidemiology, Risk factors

## Abstract

Substantial evidence has linked endocrine-disrupting chemicals and other compounds to increased risk of elevated adiposity and adverse metabolic outcomes. Individuals may be particularly susceptible to these metabolism-disrupting chemicals or compounds (MDC) during sensitive periods. The menopausal transition is a sensitive window marked by increased risk of obesity, metabolic syndrome, and cardiovascular disease that continues into the postmenopausal period; however, evidence on modifiable risk factors, such as MDC exposure, in perimenopausal individuals is limited. Therefore, we conducted a review to understand the breadth of literature evaluating relationships of MDC exposure with metabolic health outcomes (e.g. body weight/composition, glycemic control, lipid profiles, blood pressure) across the menopausal transition. We identified 28 studies using data from four U.S.-based cohorts of midlife women. Overall, studies suggest that phthalates and other nonpersistent chemicals (7 studies), per- and polyfluoroalkyl substances (7 studies), persistent organic pollutants (3 studies), heavy metals (5 studies), and air pollutants (6 studies) were associated with increases in body fat, unfavorable adipokine profiles, adverse lipid profiles, and/or higher risk of type 2 diabetes and hypertension. Several studies identified differences by race/ethnicity. Although few studies stratified by menopause status, some results suggest that perimenopause may be a sensitive window of exposure to MDCs. Additional data are needed to identify susceptible windows of exposure during midlife, particularly in more diverse populations. Future research should consider examining cumulative exposure to multi-pollutant mixtures and identifying susceptible populations and mitigation strategies during this period.

## Introduction

### Endocrine-disrupting chemicals as metabolism disruptors

Endocrine-disrupting chemicals represent a diverse class of chemicals characterized by their ability to interfere with any aspect of hormone action, and may influence human health depending on dose, duration of exposure, and timing during critical or sensitive time periods of the life course [1]. A large body of evidence also links endocrine-disrupting chemicals and other compounds to increased risk of elevated adiposity and metabolic outcomes, and substantial effort has been aimed at developing screening and testing methods to identify and characterize these metabolism-disrupting chemicals or compounds (MDC) [2, 3].

MDCs include a wide range of synthetic and natural compounds such as plasticizers, preservatives, flame retardants, pesticides, heavy metals, and air pollutants. An overview of select MDCs and their potential mechanisms of action is presented in Table 1. Chemicals used in consumer products or released as industrial byproducts can be classified as persistent or nonpersistent chemicals based on their half-life and resistance to degradation. Persistent chemicals, such as per- and polyfluoroalkyl substances (PFAS), have half-lives ranging from months to years and can persist in the environment and in the human body for many years after being banned from production. Conversely, nonpersistent chemicals, such as phthalates, have short half-lives in humans and are readily excreted in urine within 24–48 h after exposure. Individuals in the U.S. are ubiquitously exposed to MDCs [4–7]—with higher urinary concentrations of some nonpersistent chemicals in women, which may reflect use of personal care products [4, 6], and higher plasma/serum concentrations of some persistent chemicals in older adults [5, 7].

**Table 1:** Overview of select metabolism-disrupting compounds.

Compound	Uses, common exposure routes	Potential mechanisms	Reviewed in
Nonpersistent chemicals			
Bisphenols (e.g., BPA)	Food packaging/lining, polycarbonate plastics	Interacts with ESRs, AR, PXR, PPARγ, GR, TR; activates toll-like receptors and JNK/NF-kB signaling pathways; mitochondrial dysfunction	[8, 138, 139]
Phthalates (e.g., DEHP)	Plasticizers found in lubricants, personal care products, paints, toys; fragrance stabilizers in fragranced products	Interacts with PPARs, ESRs, PXR/CAR; forms reactive oxygen species	[8, 138, 139]
Parabens	Preservatives in personal care products	Interacts with ESRs, PPARγ, TR; forms reactive oxygen species	[139, 140]
Persistent chemicals			
Non-dioxin-like polychlorinated bisphenyls (PCB)	Plasticizers in adhesives, paints, plastics (banned in 1979); fish, meat, dairy	Interacts with PXR/CAR, PPARs; induces SREBP1c expression	[141]
Dioxins, dioxin-like PCBs	Byproducts of industrial processes and waste incineration; fish, meat, dairy	Interacts with AhR, PPARγ, and ESRs	[8, 141]
Organocholorine pesticides (OCP) (e.g., HCB, DDT)	Pesticides and plasticizers (DDT banned in 1970s); contaminated fish, dairy, high-fat foods	Interacts with ESRs, AR, PXR/CAR	[8, 139]
Per- and polyfluoroalkyl substances (PFAS) (e.g., PFOA)	Waterproof, greaseproof, and non-stick food packaging and consumer products; fire-fighting foams; contaminated water, fish, and other foods	Interacts with ESRs, PPARs, AR, PXR/CAR; inhibits HNF4A expression; disrupts cell membrane lipid bilayer; disrupts gut microbiome SCFA metabolism	[8, 139, 142]
Heavy metals			
Arsenic	Pesticides, mining, fracking; drinking water, soil, seafood, rice, vegetables, fruit, meat, poultry	Interacts with AhR, ESR; forms reactive oxygen species; binds to thiol and amine groups on proteins, inhibiting enzymes and leading to endoplasmic reticulum stress and mitochondrial dysfunction	[15, 143]
Cadmium	Byproduct of mining and waste incineration; cigarette smoking; contaminated crops		
Mercury	Byproduct of mining, waste incineration, industrial processes; seafood		
Lead	Paint (banned in 1978), drinking water from lead-based plumbing (banned in 1986)		
Air pollution			
Particulate matter, noxious gases (e.g., CO, NO_2_, SO_2_), ozone	Industrial processes, traffic emissions, fossil fuel and wood combustion, road dust, energy production	Inflammation, oxidative stress, and stress hormones	[144]

*AHR* aryl hydrocarbon receptor, *AR* androgen receptor, *BPA* bisphenol A, *CAR* constitutive androstane receptor, *CO* carbon monoxide, *DDT* dichlorodiphenyltrichloroethane, *DEHP* di(2-ethylhexyl) phthalate, *ESR* estrogen receptor, *GR* glucocorticoid receptor, *HCB* hexachlorobenzene, *JNK* c-Jun N-terminal kinase, *NF-κB* nuclear factor kappa-light-chain-enhancer of activated B cells, *NO*₂ nitrogen dioxide, *PCB* polychlorinated biphenyl, *PFAS* per- and polyfluoroalkyl substances, *PFOA* perfluorooctanoic acid, *PPAR* peroxisome proliferator-activated receptor, *PXR* pregnane X receptor, *SCFA* short-chain fatty acids, *SREBP1c* sterol regulatory element-binding protein 1c, *SO*₂ sulfur dioxide, *TR* thyroid hormone receptor.

Proposed pathways by which MDCs may lead to adverse metabolic health include altered function or number of β-cells or α-cells in the pancreas, impaired insulin signaling, increased adipogenesis or impaired adipocyte function, increased oxidative stress and inflammation, disrupted metabolic signaling, altered appetite signaling or energy homeostasis, and several others (e.g., gut function, microbiome, and circadian rhythm) [2]. Many MDCs interact with nuclear receptors involved in metabolic signaling and regulation [8], including: estrogen receptors (adipocyte differentiation/function, glucose transport/metabolism, β-oxidation) [9]; thyroid receptors (lipolysis, cholesterol synthesis/excretion) [10]; glucocorticoid receptors (gluconeogenesis, β-oxidation) [11]; peroxisome proliferator-activated receptors (PPARα: β-oxidation, inflammation, lipoprotein metabolism; PPARβ: β-oxidation, insulin sensitivity, PPARγ; adipogenesis, lipid storage, inflammation, insulin sensitivity) [12]; pregnane X receptor and constitutive androstane receptor (gluconeogenesis, β-oxidation, lipogenesis, inflammation, sex steroid hormone production, PPAR activation) [13]; and aryl hydrocarbon receptor (lipogenesis, glucose homeostasis, hepatic circadian rhythm, estrogen receptor signaling pathways, sex steroid hormone production and degradation, PPAR inhibition) [14]. MDCs may also influence metabolic health independent of nuclear receptors, such as through mitochondrial dysfunction and oxidative stress. For example, heavy metals disrupt the redox balance by generating reactive oxygen species and inhibiting enzymes involved in antioxidant pathways (e.g., glutathione reductase) [15].

### MDC exposure across the lifespan

Extensive research to date has focused on exposure to MDCs during fetal development and early life. In epidemiological studies, *in utero* exposure to MDCs has been associated with increased risk of obesity in childhood [16, 17], but evidence has been inconsistent and may be sex-specific. Fewer studies have focused on other sensitive windows beyond early life, such as periods of hormonal shifts during puberty and pregnancy. Although studies are limited, MDC exposure during adolescence has been associated with earlier onset of puberty [18] and may be associated with adverse lipid profiles [19, 20]. During pregnancy, MDC exposure has been associated with increased risk for gestational diabetes [21, 22] and hypertensive disorders of pregnancy [22–24], as well as higher weight retention up to 1–7 years postpartum [22, 24]; however, data on other metabolic outcomes postpartum are limited [22]. Beyond the reproductive years, menstruating individuals experience another hormonal shift during the menopausal transition, which has been understudied compared to earlier life stages and may represent another sensitive window for MDC exposure [25].

### Perimenopause and the menopausal transition

The menopausal transition is defined by variable length in menstrual cycles, approximately 2–8 years prior to the final menstrual period [26]. Menopause—the cessation of the menstrual cycle—is defined as occurring 12 months after the final menstrual period [26]. In the U.S., the typical age of menopause is approximately 50 years [27], with the postmenopausal period representing up to 30–40% of an individual’s life. Perimenopause is the period beginning with the menopausal transition and ending 1 year after the final menstrual period [26], with the onset of related symptoms (e.g., hot flashes) starting as early as 10 years prior to menopause [28]. The underlying pathophysiology of perimenopause is a rapid decline in ovarian follicle reserves, leading to changes in ovarian follicle activity and hormones [29].

Perimenopause is a sensitive window marked by increased risk of obesity, metabolic syndrome, and cardiovascular disease that continues into the postmenopausal period [30, 31]. Evidence suggests that this period marks a reversal of high-density lipoprotein cholesterol’s protective role in cardiovascular disease [32]. Although aging in general is associated with weight gain and adverse metabolic health, studies have shown that earlier onset of menopause increases cardiometabolic risk [33], and ovarian aging drives the risk of metabolic syndrome independent of biological aging [34], with the greatest increase in severity occurring during the pre- and perimenopausal period [35]. Findings from a meta-analysis suggest that increasing total body fat in midlife women is likely driven by biological age, but that increasing central adiposity is more specific to menopause [36]. Although studies do not support a role of ovarian aging in the development of type 2 diabetes beyond biological aging [37], menopause-specific increases in central adiposity may contribute to the development of insulin resistance and other metabolic outcomes. More recently, data suggest menopause, independent of age, may also alter gut microbial diversity and bacterial abundance [38], and these menopause-specific alterations have been associated with adverse metabolic health [39].

Perimenopause additionally marks changes in adipose tissue metabolism that may predispose postmenopausal women to weight gain and insulin resistance, including lipid spillover to visceral adipose tissue, and metabolic changes in subcutaneous adipose tissue (e.g., adipocyte hypertrophy, inflammation, hypoxia, and fibrosis) and visceral adipose tissue (e.g., adipocyte hypertrophy, immune cell infiltration, fibrosis, and increased PPARγ expression) [40]. The expression of estradiol-producing enzymes (e.g., 17β-hydroxysteroid dehydrogenase), a potential target of MDCs, was also shown to increase in subcutaneous and visceral adipose tissue in postmenopausal women compared to premenopausal women [41].

The increased risk of metabolic outcomes during perimenopause is likely due to changes in hormones. The perimenopausal period is first marked by declining inhibin B—a peptide produced by granulosa cells of ovarian follicles—which leads to a rise in follicle-stimulating hormone (FSH). FSH signals the ovaries to release estradiol, which begins to decline later in perimenopause as follicle reserves decrease. FSH plays a critical role in hepatic cholesterol synthesis as well as adipogenesis via PPARγ activation [42], and estrogens, acting via estrogen receptors and estrogen receptor-independent pathways, are involved in energy expenditure, fat redistribution, insulin sensitivity, and mitochondrial function [43]. In midlife women, higher FSH concentrations are associated with increasing adiposity [44] and adverse lipid profiles [45], and an increasing ratio of testosterone to estradiol predicted higher risk of metabolic syndrome [46]. Concentrations of both FSH and estradiol have also been associated with unfavorable adipokine profiles [47, 48] as well as shifts in the gut microbiome [38]. The trajectory of FSH and estradiol during perimenopause varies across individuals [49], and concentrations may be influenced by factors such as body mass index (BMI) and race/ethnicity [49], as well as MDCs [25]. Hormonally-mediated changes in adipose tissue and metabolism in perimenopause may be compounded by lifestyle changes during this period (e.g., dietary patterns, physical activity, sleep, medications) [50]. Together, these factors may contribute to increased vulnerability to MDCs, which may exacerbate oxidative stress, interfere with compensatory mechanisms such as peripheral estrogen production, or disrupt metabolic signaling via nuclear receptors.

Although data suggest the perimenopausal period is a sensitive window for adverse metabolic health, evidence for prevention strategies in this population is limited. For example, data on exogenous hormone interventions to prevent risk of cardiovascular disease suggest that timing relative to menopause (within 10 years) is critical [51]; however, no studies have been conducted among perimenopausal individuals to inform potential benefits of earlier intervention [30]. Additional data in perimenopause are needed to identify risk factors, such as MDC exposure, and interventions to improve metabolic health. Therefore, the objective of this review was to understand and synthesize the breadth of literature evaluating relationships of MDCs (nonpersistent chemicals, persistent chemicals, heavy metals, air pollution) with obesity and metabolic outcomes during perimenopause. We then discuss research gaps and future directions for informing strategies to mitigate MDC exposure during this sensitive window.

## Methods

We reviewed the literature for studies assessing MDC exposure during the perimenopausal period with measures of body composition, adiposity, and metabolic health outcomes, including insulin resistance and type 2 diabetes, blood pressure and hypertension, lipid profiles, and markers of inflammation and metabolic disturbance (e.g., adipokine levels). Specifically, we focused on data from cohort studies of midlife women in which most participants at baseline were pre- or perimenopausal.

We developed a search strategy in PubMed with title/abstract and medical subject heading terms for MDCs (e.g., phthalates, PFAS, organochlorine pesticides, polychlorinated biphenyls [PCB], dioxins, bisphenols, air pollution, heavy metals), perimenopause (e.g., perimenopause, menopause, midlife women), and metabolic outcomes (e.g., metabolic syndrome, diabetes, insulin, adiposity, obesity, blood pressure, hypertension, triglycerides, adipokines) (Supplementary Table 1). The search strategy identified 297 abstracts, which we reviewed for relevance. We retrieved full texts and extracted data from 28 included studies. Included studies were conducted within four U.S. cohorts. Two of these cohorts specifically focused on the perimenopausal period: (1) Midlife Women’s Health Study (MWHS; 45–54 years; 100% pre- and perimenopausal) in Baltimore; (2) the Study of Women’s Health Across the Nation (SWAN; 45–56 years; ~70% pre- and perimenopausal), a U.S.-representative cohort of pre-, peri-, and postmenopausal women. Other studies were conducted in two cohorts of registered nurses from across the U.S., which included a broader range of ages: (3) the Nurses’ Health Study (NHS; 53–79 years; >99% postmenopausal); and (4) NHSII (32–52 years; ~65% pre- and perimenopausal). In addition to included studies, other studies in postmenopausal women (e.g., the Women’s Health Initiative cohort) or studies among both midlife and younger women stratified by menopause status (e.g., women ≥18 years in the U.S. National Health and Nutrition Examination Survey [NHANES]) were discussed to provide additional context. Included studies were synthesized narratively and in tables and presented separately by exposure, namely nonpersistent chemicals (e.g., phthalates, phenols [bisphenols, parabens, others]), persistent chemicals (e.g., PFAS, persistent organic pollutants (POP) such as PCB), and other compounds previously shown to have metabolism-disrupting properties (e.g., heavy metals, air pollution).

## Results

### Nonpersistent chemicals

An overview of studies assessing nonpersistent chemicals and metabolic health among perimenopausal women is presented in Table 2, and results from these studies as well as supporting data from other studies are synthesized below.

**Table 2:** Overview of studies assessing associations of non-persistent chemicals with adiposity and metabolic health outcomes during the perimenopausal period.

Study ID	Cohort	Age; % pre- and perimenopausal	*N*	Follow-up	Exposure	Outcome	Stratified analyses	Summary of results
Haggerty [52]	MWHS;	45–54 years; 100%	524	1 year	Phthalates^c^	BMI	Menopause transition status	Overall: none associated with BMI By menopause transition status: MiBP, MEP, DEHP metabolites (MEHP, MEHHP, MEOHP, ΣDEHP) associated with increase in BMI in women transitioning from peri- to postmenopause; MBP, MEP associated with decrease in BMI in women who stayed perimenopausal; no associations in women who stayed premenopausal or transitioned from pre- to perimenopause
Song [53]	NHS, NHSII	NHSII 32–52 years, ~65%; NHS 53–79 years, <1%; overall 32–79 years, 40%	977	10 years	Phthalates, BPA^a^	Weight gain	Age (≥53 years), BMI	Overall: BPA, phthalic acid, MBzP, ΣButyl phthalates, total phthalates associated with increased weight gain By age: no differences by age By BMI (<25 kg/m^2^): no differences by BMI
Peng [55]	SWAN	45–56 years; 71%	1369	17 years	Phthalates^a^	fat mass, % fat, weight	BMI	Overall: none associated with weight; MEHP, MEHHP, MEOHP, MECPP, ΣDEHP associated with higher fat mass; MEHP, MEHHP, MEOHP, MECPP, ΣDEHP, MBzP, MCOP, MCPP associated with increased % fat By BMI: BMI 25-30 or ≥ 30 kg/m^2^: no or few metabolites associated with weight, fat mass, % fat; BMI<25 kg/m^2^: MEP, MBzP, MCPP associated with higher weight; MEP, MnBP, MiBP MEHP, MEHHP, MEOHP, MECPP, ΣDEHP, MBzP, MCOP, MCPP associated with increased fat mass and % fat
Lee [56]	SWAN	42–52 years; 41%	1200	Cross-sectional	Phenols^a^	Adipokines	Race/ethnicity, BMI	Overall: 2,4-DCP associated with higher adiponectin; methyl paraben associated with lower leptin; BPF, methyl paraben, and ethyl paraben associated with higher soluble leptin receptor (favorable profile) By race/ethnicity: BPF, 2,4-DCP, methyl paraben, ethyl paraben associated with more favorable profile in White women; methyl paraben and phenol mixture associated with higher leptin in Black women (unfavorable profile) By BMI (<30 kg/m^2^): No differences by BMI
Peng [58]	SWAN	45–56 years; 71%	1308	6 years	Phthalates^a^	T2D, insulin resistance, fasting glucose	Race/ ethnicity	Overall: none associated with T2D; MnBP, MiBP, MEHP, MEHH, MEOHP, MECPP, ΣDEHP, MBzP associated with increased fasting glucose; DEHP metabolites (MEHHP, MEOHP, MECPP, ΣDEHP) associated with higher insulin resistance and MEP associated with lower insulin resistance By race/ethnicity: MiBP, MBzP, MCOP, MCNP, MCPP associated with higher incident T2D in White women, but not Black or Asian women; MiBP, MEHP, MEHH, MEOHP, MECPP, ΣDEHP associated with increased fasting glucose in White women; MBzP associated with increased fasting glucose in Black women; MEHHP, MECPP, ΣDEHP associated with increased fasting glucose in Asian women
Sun [59]	NHS, NHSII	NHSII 32–52 years, ~65%; NHS 53–79 years, <1%; overall 32–79 years, 40%	1941	6–11 years	Phthalates, BPA^a^	T2D	Cohort	Overall: MECPP and phthalic acid associated with higher odds of T2D NHS (≥53 years): none associated with T2D NHSII (<53 years): BPA, MBP, MiBP, total phthalates associated with higher odds T2D
Lee [57]	SWAN	45–56 years; 71%	1299	17 years	Phenols^b^	T2D	none	Overall: Third vs. first tertile: methyl paraben, propyl paraben, 2,5-DCP, Benzophenone-3 associated with lower incident T2D; Second vs. first tertile: BPA and 2,4-DCP associated with higher incident T2D; qGcomp mixture not associated with T2D

*BMI* body mass index, *BKMR* Bayesian kernel machine regression, *BPA* bisphenol A, *BPF* bisphenol F, *DEHP* di(2-ethylhexyl) phthalate, *MBP* mono-n-butyl phthalate, *MBzP* monobenzyl phthalate, *MCOP* monocarboxyisononyl phthalate, *MECPP* mono(2-ethyl-5-carboxypentyl) phthalate, *MEHP* mono(2-ethylhexyl) phthalate, *MEHHP* mono(2-ethyl-5-hydroxyhexyl) phthalate, *MEOHP* mono(2-ethyl-5-oxohexyl) phthalate, *MEP* monoethyl phthalate, *MiBP* monoisobutyl phthalate, *MWHS* Midlife Women’s Health Study, *NHS* Nurses’ Health Study, *NHSII* Nurses’ Health Study II, *qGcomp* quantile g-computation, *SWAN* Study of Women’s Health Across the Nation, *T2D* type 2 diabetes, *2,4-DCP* 2,4-dichlorophenol, *2,5-DCP* 2,5-dichlorophenol.

^a^1 spot urine sample used for exposure assessment.

^b^2 spot urine samples used for exposure assessment.

^c^2-4 spot urine samples used for exposure assessment.

#### Phthalates and adiposity

Studies suggest that exposure to phthalates during perimenopause may be associated with increases in body fat over time. Overall, phthalates were not associated with 1-year change in BMI among pre- and perimenopausal women in MWHS [52]. However, in stratified analyses, several metabolites of di-(2-ethylhexyl) phthalate and their sum, as well as monoisobutyl and monoethyl phthalates (metabolites of diisobutyl and diethyl phthalates, respectively), were associated with increased BMI among women who transitioned from peri- to postmenopause from baseline to follow-up 1 year later [52], suggesting a sensitive window of exposure during late perimenopause when shifts in hormones (e.g., decreases in estradiol) are particularly notable. These findings were generally supported by other studies assessing weight gain, including a study in NHS and NHSII [53] and a study among postmenopausal women in the Women’s Health Initiative cohort [54]. However, BMI and body weight may not be ideal measures of body fat in older ages as muscle mass decreases, and the limited follow-up period in the MWHS study prevented assessment of longer-term changes in postmenopause. In a study of pre-, peri-, and postmenopausal women from the SWAN cohort [55], most phthalate metabolites were associated with a 5-year increase in fat mass and percent body fat, but not body weight, and associations were strongest among women with BMI < 25 kg/m^2^. As noted by authors, results were attenuated when assessing exposure 3 years later, when more participants may have transitioned to being postmenopausal, which could indicate a sensitive window during pre- and perimenopause.

#### Nonpersistent chemicals and metabolic outcomes

Evidence of the roles of nonpersistent chemicals in metabolic outcomes among midlife women suggests that findings may differ by chemical exposure and race/ethnicity. In a cross-sectional analysis of SWAN (described earlier), some individual phenols (bisphenol F, 2,4-dichlorophenol, methyl-, ethyl-, and propylparabens) were associated with more favorable adipokine levels (i.e., higher adiponectin, higher leptin receptor, and/or lower leptin) in White women, whereas the phenol mixture (driven by methylparaben) was associated with unfavorable adipokine levels (i.e., higher leptin) in Black women, but not White or Asian women [56]. In a longitudinal analysis of the same population, some phenols (bisphenol A, 2,4-dichlorophenol) were nonmonotonically associated with a higher risk of type 2 diabetes, whereas parabens (ethyl- and propylparabens) and other phenols (2,5-dichlorophenol, benzophenone-3) were associated with lower risk of type 2 diabetes [57]; however, stratified analyses were not conducted to detect potential differences by race/ethnicity. Exposure to phthalates was associated with a higher incidence of type 2 diabetes among White women but not Black or Asian women in the SWAN cohort [58]. Similarly, in the NHSII cohort of majority White participants, bisphenol A and several phthalate metabolites were also associated with higher risk of type 2 diabetes [59].

In addition to included studies, other studies assessing exposures in postpartum (with midlife outcomes) [60] or in majority postmenopausal women (e.g., [59, 61, 62]) suggest that nonpersistent chemicals are associated with improved metabolic health or not associated with metabolic outcomes, whereas findings stratified by menopause status are inconsistent in population-based studies among a wide range of ages (e.g., ≥18 years) (e.g., [63, 64]), highlighting the need for more studies evaluating MDC exposure during the perimenopausal period.

### Persistent chemicals

An overview of studies assessing persistent chemicals and metabolic health among perimenopausal women is presented in Table 3, and results from these studies as well as supporting data from other studies are synthesized below.

**Table 3:** Overview of studies assessing associations of persistent chemicals with adiposity and metabolic health outcomes during the perimenopausal period.

Study ID	Cohort	Age; % pre- and perimenopausal	*N*	Follow-up	Exposure	Outcome	Stratified analyses	Summary of results
Ding [65]	SWAN	45–56 years; 71%	1381	19 years	PFAS	Weight, WC, fat mass, % fat	none	Overall: PFOS, n-PFOS, sm-PFOS, EtFOSAA, MeFOSAA associated with higher weight, WC, body fat, and % fat, and with accelerated increases in weight, WC, and body fat
Ding [66]	SWAN	45–56 years; 71%	1245	3 years	PFAS	Adipokines	BMI, menopause status	Overall: Independent of WC, PFOS, n-PFOS, sm-PFOS, n-PFOA, EtFOSAA, MeFOSAA, and BKMR mixture (highest contributors: sm-PFOS, *n*-PFOA, EtFOSAA) associated with higher leptin and free leptin index; and none associated with sOB-R, adiponectin, or HMW adiponectin By BMI: Associations with higher leptin and FLI were stronger among women with BMI ≥ 25 vs. <25 kg/m^2^ (*p*-interaction <0.05); PFNA associated with lower adiponectin and HMW adiponectin in women with BMI < 25 but not ≥ 25 kg/m^2^ (*p*-interaction = 0.02) By menopause status: Associations with higher leptin and FLI were stronger among pre/early perimenopausal women vs. late peri/postmenopausal women (*p*-interaction < 0.01); n-PFOA associated with lower sOB-R, adiponectin, and HMW adiponectin in pre/early perimenopausal women but not late peri/postmenopause (*p*-interaction < 0.05)
Park [67]	SWAN	45–56 years; 70%	1237	17 years	PFAS	T2D	Race/ ethnicity	Overall: PFOA, PFHxS, MeFOSAA, total PFOS, n-PFOS, and sum of four PFAS (n-PFOA, PFNA, PFHxS, total PFOS) associated with higher risk of T2D; qGcomp mixture associated with higher risk of T2D (highest contributor: MeFOSAA) By race/ethnicity: Associations with higher risk of T2D larger in White women compared to Black or Asian women, except MeFOSAA associated with higher risk of T2D in Black women compared to White women (*p*-interaction < 0.10)
Sun [68]	NHSII	32–52 years; 68%	1586	11–16 years	PFAS	T2D	Time of diabetes diagnosis, AHEI	Overall: PFOS and PFOA associated with higher risk of T2D By time of diabetes diagnosis or AHEI: no interactions
Kang [71]	SWAN	45–56 years; 63%^a^	1130	17 years	PFAS	Lipids	none	Overall:^b^ several PFAS associated with higher TC and LDL (n-PFOS, sm-PFOS, PFDA, PFUnDA, PFHxS, MeFOSAA) and lower HDL (sm-PFOS); and nonmonotonically associated with higher TC (PFNA) and LDL (PFNA, EtFOSAA) and lower HDL (EtFOSAA); other PFAS associated with lower TC (EtFOSAA) and nonmonotonically associated with lower TC (n-PFOA) and higher HDL (MeFOSAA); none associated with TG; PFAS mixture using K-means clustering associated with higher TC and LDL but not HDL or TG; Few PFAS associated with rate of change in lipid biomarker
Ding [69]	SWAN	45–56 years; 72%	1058	17 years	PFAS	HTN	Menopause status	Overall: PFOS, n-PFOS, sm-PFOS, n-PFOA, EtFOSAA, MeFOSAA, and qGcomp mixture associated with higher incidence of HTN; PFOS, n-PFOS, sm-PFOS, n-PFOA, EtFOSAA associated with shorter time to HTN development By menopause status: MeFOSAA associated with higher risk of HTN in pre- and postmenopausal but not perimenopausal women
Ding [70]	SWAN	45–56 years; 72%	1058	17 years	PFAS	HTN	none	PFAS exposure (PFOS, n-PFOS, EtFOSAA, MeFOSAA, qGcomp mixture) mediated racial disparities in HTN
Grant-Alfieri [75]	SWAN	45–56 years; 63%^1^	838–1040	17 years	POPs	T2D	none	Overall: HCB and PCB-123 nonmonotonically associated with higher risk of T2D
Wu [78]	NHS	43–69 years; not reported	1095	9 years	POPs	T2D	none	Overall: HCB associated with higher risk of T2D
Zong [76]	NHSII	32–52 years; 68%	1586	11–16 years	POPs	T2D	Age, breastfeeding duration, weight change, current BMI	Overall: β-HCH, p,p’-DDE, PCB-TEQ, PCBs individually (74, 105, 138, 157, 167), group of antiestrogenic, immunotoxic, dioxin-like moderately persistent PCBs (74, 105, 118, 156, 157), and PCBs with ≤4 or 5 chlorines associated with higher risk of T2D By weight change: HCB, β-HCH, PCB-TEQ associated with higher risk of T2D in women with stable body weight from enrollment to blood draw (*p*-interaction < 0.05) By age: β-HCH associated with higher risk of T2D in women 40–50 years and >50 years (but not <40 years) (p-interaction = 0.07) By BMI: β-HCH and PCB-TEF associated with higher risk of T2D among women with BMI < 25 or >30 but not 25–30 kg/m^2^ (*p*-interaction <0.05) By breastfeeding duration: no significant interaction

*AHEI* Alternative Healthy Eating Index, *BKMR* Bayesian kernel machine regression, *BMI* body mass index, *EtFOSAA* 2-(N-Ethyl-perfluorooctane sulfonamido) acetic acid, *FLI* free leptin index, *HCB* hexachlorobenzene, *HDL* high density lipoprotein cholesterol, *HTN* hypertension, *LDL* low density lipoprotein cholesterol, *MeFOSAA* 2-(N-methyl-perfluorooctane sulfonamido) acetic acid, *NHS* Nurses’ Health Study, *NHSII* Nurses’ Health Study II, *n-PFOA* n-perfluorooctanoic acid (linear isomers), n-*PFOS* n-perfluorooctane sulfonate (linear isomers), *PCB* polychlorinated biphenyl, PCB-TEF: polychlorinated biphenyl-toxic equivalency factor, *PCB-TEQ* polychlorinated biphenyl-toxic equivalency quotient, *PFAS* per- and polyfluoroalkyl substances, *PFHxS* perfluorohexane sulfonate, PFOS: perfluorooctane sulfonate, *p,p’-DDE* 1,1-dichloro-2,2-bis(p-chlorophenyl)ethylene, *POP* persistent organic pollutant, *sOB-R* soluble leptin receptor, *sm-PFOS* sum of perfluorooctane sulfonate isomers (sum of branched isomers), *SWAN* Study of Women’s Health Across the Nation, *T2D* type 2 diabetes, *WC* waist circumference, *β-HCH* beta-hexachlorocyclohexane.

^a^Percentage represents pre- and early perimenopause, while late perimenopause and postmenopause were grouped together.

^b^Latent class growth models were used to categorize lipid trajectories (high vs. low).

#### PFAS and adiposity/adipokines

Studies suggest that PFAS may be associated with increasing body fat and waist circumference, as well as less favorable adipokine concentrations, particularly in early perimenopause. In SWAN, several PFAS, including perfluorooctane sulfonic acid (PFOS), 2-(N-ethyl-perfluorooctane sulfonamido) acetic acid (EtFOSAA), and 2-(N-methyl-perfluorooctane sulfonamide) acetic acid (MeFOSAA), were associated with higher indicators of body fat (body weight, waist circumference, body fat, percent body fat) and with accelerated increases of these measures over time [65]. In a 3-year follow-up analysis in SWAN, individual PFAS (PFOS, EtFOSAA, MeFOSAA, perfluorooctanoic acid [PFOA], perfluorononanoic acid [PFNA]) and PFAS mixtures were associated with unfavorable adipokine profiles (i.e., higher leptin and free leptin index [ratio of leptin to leptin receptor]), independent of waist circumference, and associations were stronger in women with BMI ≥ 25 kg/m^2^ (women with overweight or obesity) [66]. The associations of PFAS with unfavorable adipokine profiles were also stronger in pre-/early perimenopausal women compared to late peri-/postmenopausal women [66], suggesting a sensitive window of exposure during early perimenopause.

#### PFAS and other metabolic outcomes

Studies suggest PFAS increases risk of type 2 diabetes and hypertension, which may differ by race/ethnicity. Once again in SWAN, higher PFAS (PFOS, PFOA, MeFOSAA, perfluorohexane sulfonic acid [PFHxS]) and PFAS mixtures were associated with greater risk of type 2 diabetes, with MeFOSAA being the greatest contributor to the overall PFAS mixture [67]. The associations between PFAS and diabetes were generally stronger among White women compared to Black or Asian women, except for MeFOSAA, which was associated with greater risk of diabetes in Black women compared to White women [67]. Similarly, in a nested case control study of predominately White women from NHSII, PFOS and PFOA were associated with higher risk of type 2 diabetes [68].

In another study conducted within SWAN, several PFAS (PFOS, PFOA, EtFOSAA) and PFAS mixtures were also associated with a higher incidence of hypertension and shorter time to development of hypertension regardless of menopause status [69]. Interestingly, MeFOSAA was associated with a higher risk of hypertension among premenopausal and natural postmenopausal women but not those in perimenopause [69]. Black women were at a higher risk of developing hypertension in the SWAN cohort compared to White and Asian women, and disparities in PFAS concentrations as a mixture explained 19.1% of the racial/ethnic disparities in hypertension [70]. In another study within the SWAN cohort, PFAS were associated with higher odds of having adverse lipid trajectories, including higher low-density lipoprotein and total cholesterol (PFOS, PFHxS, MeFOSAA, perfluoroundecanoic acid [PFUnDA], perfluorodecanoic acid [PFDA]) and lower high-density lipoprotein cholesterol (PFOS) [71]; however, analyses did not stratify by race/ethnicity.

In contrast to results from SWAN and NHSII, studies in Sweden (menopause status not reported or considered in analyses) reported that PFAS exposure was associated with lower fat mass (50 years; cross-sectional) [72], lower prevalence of type 2 diabetes (45–75 years; cross-sectional) [73], and lower fasting glucose concentrations (70 years; 10 year follow-up) [73] in women, but not men. However, the cross-sectional design limits interpretability, and the inclusion of a large range of postmenopausal ages suggests that the role of PFAS in increased diabetes risk may be more relevant to earlier exposure during perimenopause.

#### Persistent organic pollutants and metabolic outcomes

While PFAS are bound to circulating proteins in the human body, POP, such as PCBs and organochlorine pesticides, accumulate in adipose tissue—suggesting that adipose tissue may be both a target of POPs and a modulator of circulating POP concentrations. For example, in a small subset of women in the SWAN cohort, increases in waist circumference were associated with lower circulating concentrations of PCB-194, a highly lipophilic compound [74], highlighting changes in body composition as a potential confounder when evaluating relationships between POPs and metabolic endpoints. In the full SWAN cohort of pre-, peri-, and postmenopausal women, few POPs at baseline were associated with risk of diabetes, except hexachlorobenzene and PCB-123, which were nonmonotonically associated with higher risk of diabetes [75]; however, analyses did not adjust for changes in body composition over time. In the NHSII cohort, several POPs including β-hexachlorocyclohexane, p,p’dichlorodiphenyldichloroethylene, individual PCB congeners, and summative PCB measures (PCB-toxicity equivalents; sum of PCBs considered antiestrogenic, immunotoxic, dioxin-like, and moderately persistent; and sum of PCBs with ≤4 or 5 chlorines) were associated with higher risk of type 2 diabetes in models adjusted for weight change and current BMI [76], with most associations being stronger in women with stable weight compared to those who gained weight.

In addition to weight gain, menopause status has been shown to impact the distribution of POPs in plasma, visceral adipose tissue, and subcutaneous adipose tissue [77], suggesting that the association between POPs and metabolic health may vary by menopause status. In NHSII, the association between β-hexachlorocyclohexane and higher risk of type 2 diabetes was stronger in older women (40–50 and >50 years) compared to younger women who may have been predominantly premenopausal (<40 years) [76]. This result is supported by several studies in older women: including plasma hexachlorobenzene and higher incidence of type 2 diabetes in the older NHS cohort of majority postmenopausal women [78], and several PCBs with higher odds of diabetes and insulin resistance in cross-sectional studies of postmenopausal women [79, 80]. In contrast, in other studies including a wide range of ages (e.g., ≥18 years), organochlorine pesticides were associated with a higher risk of type 2 diabetes in pre-/perimenopausal women, but not postmenopausal women (China) [81], and organophosphate esters were not consistently associated with metabolic syndrome and its components regardless of menopause status (U.S. NHANES) [82].

### Heavy metals

Several studies conducted within the SWAN cohort suggest that heavy metals may be associated with adverse metabolic health outcomes, including type 2 diabetes and metabolic syndrome (Table 4). In SWAN, barium and lead were associated with higher risk of abdominal obesity [83], and cadmium, cesium, and lead were associated with an unfavorable adipokine profile (lower adiponectin or lower soluble leptin receptor) [84]. Several studies within SWAN have linked arsenic with metabolic syndrome [83] and impaired glucose homeostasis, including insulin resistance and lower β-cell function [85], impaired fasting glucose [83], and type 2 diabetes [86]. Arsenic, cadmium, and nickel were associated with a higher risk of high blood pressure in an analysis focused on metabolic syndrome [83], and in another SWAN study, arsenic, mercury, and lead were associated with increases in systolic and diastolic blood pressure over time, whereas cadmium was associated with increases in systolic blood pressure in never smokers only [87]. In addition to nonessential heavy metals, deficiency or excess of essential metals can result in adverse health [88]. For example, molybdenum was associated with a lower risk of abdominal obesity [83] and a favorable adipokine profile [84], whereas other essential metals, such as cobalt, copper, and zinc, were associated with a higher risk of abdominal obesity, high blood pressure, metabolic syndrome, and/or type 2 diabetes [83, 85, 86].

**Table 4:** Overview of studies assessing associations of heavy metals with adiposity and metabolic health outcomes during the perimenopausal period.

Study ID	Cohort	Age; % pre- and perimenopausal	N	Follow-up	Exposure	Outcome	Stratified analyses	Summary of results
Wang [84]	SWAN	45–56 years; 70%	1228	3 years	Urinary metals	Adipokines	none	Overall**:** In AENET models, molybdenum associated with higher HMW-adiponectin, lower leptin, higher sOB-R (favorable profile); cadmium associated with lower HMW-adiponectin, cesium and lead associated lower sOB-R (unfavorable profile)
Wang [85]	SWAN	45–56 years; 69%	1262	17 years	Urinary metals	HOMA-IR, HOMA-B	none	Overall: In AENET models, zinc associated with faster increase in HOMA-IR, arsenic is associated with a faster decline in HOMA-B
Wang [86]	SWAN	45–56 years; 70%	1237	17 years	Urinary metals	T2D	none	Overall: Arsenic, lead, zinc associated with higher risk of T2D
Wang [83]	SWAN	45–56 years; 71%	947	17 years	Urinary metals	MetS	Race/ethnicity; Menopause status	Overall: Arsenic, cobalt, zinc associated with higher risk of MetS; several heavy metals associated with components of MetS including higher risk of high blood pressure (arsenic, cobalt, zinc, cadmium, nickel), impaired fasting glucose (arsenic, cobalt, zinc, barium, nickel), high triglycerides (zinc); abdominal obesity (cobalt, zinc, barium, copper, lead); others associated with lower risk of high triglycerides (arsenic) and abdominal obesity (molybdenum) By race/ethnicity: Cadmium associated with lower risk of MetS in Black women but not White and Asian women, manganese associated with higher risk in Asian women, nickel and antimony associated with higher risk in White women (*p*-interaction < 0.10) By menopause status: Manganese, cadmium, mercury, molybdenum, lead, tin associated with a higher risk of MetS in postmenopausal women compared to premenopausal women (*p*-interaction < 0.10)
Wang [87]	SWAN	45–56 years; 71%	1317	17 years	Urinary metals	HTN, SBP, DBP	Smoking status	Overall: Arsenic, mercury, lead associated with increases in SBP and DBP over time By smoking status (cadmium only): cadmium is associated with increased SBP in never smokers only

*AENET* adaptive elastic net, *DBP* diastolic blood pressure, *HMW*high molecular weight, *HOMA-IR* homeostatic model assessment for insulin resistance, *HOMA-B* homeostatic model assessment for β-cell function, *HTN* hypertension, *MetS* metabolic syndrome, *SBP* systolic blood pressure, *sOB-R* soluble leptin receptor, *SWAN* Study of Women’s Health Across the Nation, *T2D* type 2 diabetes.

Studies in SWAN evaluating associations of heavy metals with metabolic health did not stratify by menopause status; however, other studies suggest that associations may differ between pre-/perimenopausal women and postmenopausal women. For example, the association between a mixture of heavy metals, especially mercury, with a higher score for 10-year CVD risk was stronger in postmenopausal women than in pre-/perimenopausal women ≥ 20 years in Korea [89]. Similarly, blood lead was associated with higher systolic and diastolic blood pressure and odds of elevated diastolic blood pressure, which was strongest in postmenopausal women (NHANES; 40–59 years) [90]. In contrast, serum mercury was associated with higher odds of hypertension only among pre-/perimenopausal women (Korea NHANES; ≥ 18 years) [91]. However, studies stratifying by menopause status combined pre- and perimenopausal women, with most including women as young as 18 years.

### Air pollution

Studies assessing air pollution and metabolic health are summarized in Table 5 and suggest that long-term exposure to air pollution increases risk for elevated adiposity, metabolic outcomes, and other risk factors for cardiovascular disease in midlife women. In the SWAN cohort, exposure to particulate matter with a diameter <2.5 μm (PM_2.5_), nitrogen dioxide (NO_2_), and ozone (O_3_) was associated with increased fat mass and percent fat mass, but decreased lean mass, especially among women with less physical activity [92]; however, differences by menopause status were not considered. Similar associations were identified in NHSII and NHS, in which PM_2.5_ and NO_2_ were associated with increased BMI [93]; associations were stronger in postmenopausal women in the younger cohort (NHSII), whereas findings did not differ by menopause status in the older cohort (NHS), potentially due to different age ranges and distribution of perimenopausal status. These findings are supported by a large representative longitudinal study in Chinese adults (≥45 years; menopause status not reported), in which long-term exposure to PM_2.5_, O_3_, and NO_2_ were associated with higher waist circumference and body weight and associations were significantly stronger in women compared to men [94]. In another study in SWAN, PM_2.5_ exposure and the mixture of PM_2.5_, NO_2_, and O_3_ were associated with an unfavorable adipokine profile, with associations stronger for O_3_ in Black women and associations stronger for NO_2_ in women with elevated waist circumference (≥88 cm) [95]; however, analyses did not stratify by menopause status.

**Table 5:** Overview of studies assessing associations of air pollution with adiposity and metabolic health outcomes during the perimenopausal period.

Study ID	Cohort	Age; % pre- and perimenopausal	*N*	Follow-up	Exposure	Outcome	Stratified analyses	Summary of results
Wang [92]	SWAN	45–56 years; 68%	1654	8 years	PM_2.5_, NO_2_, O_3_	Weight, BMI, WC, fat mass, % fat, lean mass	Physical activity, race/ethnicity	Overall: PM_2.5_ and NO_2_ associated with increased fat mass and % fat, decreased lean mass, faster increases in WC, slower increases in fat mass and percent body fat; O_3_ associated with increased % fat, decreased lean mass, faster increases in weight, BMI, and WC, slower increases in fat mass and % fat By physical activity: Associations of PM_2.5_ and NO_2_ with body composition were weaker among participants who engaged in more physical activity (*p*-interaction < 0.01) By race/ethnicity: Associations of PM_2.5_ and NO_2_ with increased fat mass and % body fat stronger in Japanese women, associations with decreased lean mass stronger in Black women; associations with body composition stronger in White women from the Michigan site compared to sites in California and Pennsylvania
Zhang [93]	NHS; NHSII	NHSII 25–42 y, ~65%; NHS 42–75 years, 10%; overall 25–75 years, 30%	194 966	28 years	PM_2.5_, NO_2_	BMI	Age, menopause, population density, physical activity, AHEI, SES	NHSII: PM_2.5_ and NO_2_ associated with increased BMI; PM_2.5_ associated with increased risk of obesity; associations between PM_2.5_ and BMI are stronger in postmenopausal women and women in the highest quintile of neighborhood SES score NHS: PM_2.5_ and NO_2_ associated with increased BMI; PM_2.5_ and O_2_ associated with increased risk of obesity; associations between PM_2.5_ and BMI were stronger in women in the lowest quintile of neighborhood SES score and AHEI but did not differ by menopause status
Wang [95]	SWAN	45–56 years; 42%	1551	3 years	PM_2.5_, NO_2_, O_3_	Adipokines	Race/ethnicity, WC	Overall: Long-term PM_2.5_ associated with lower HMW adiponectin, and BKMR mixture associated with lower HMW adiponectin and higher leptin (unfavorable profile) By strata: O_3_ associated with higher leptin and lower sOB-R (*p*-interaction <0.001 and 0.04); NO₂ associated with lower HMW adiponectin levels in participants with WC ≥ 88 cm (*p*-interaction = 0.01)
Wu [97]	SWAN	45–56 years; not reported	2289	6 years	PM_2.5_	Lipid profile	Menopause status, dyslipidemia	Overall: Long-term PM_2.5_ associated with higher Lp(a), ApoB, ApoB/ApoA1 ratio and lower HDL and ApoA1 By dyslipidemia: Long-term PM_2.5_ associated with higher LDL, Lp(a), ApoB in women without dyslipidemia and lower ApoA1 in women with dyslipidemia By menopause status: Long-term PM_2.5_ associated with higher LDL, Lp(a), ApoB, ApoB/ApoA1 and lower HDL and ApoA1 in perimenopausal but not postmenopausal women
Wu [96]	SWAN	45–56 years; not reported	2306	5 years	CO, NO_2_, SO_2_	Lipid profile	none	Overall: Short-term CO associated with increased HDL, APoA1, and reduced TG; Short-term NO_2_ associated with reduced TG; long-term NO_2_ associated with reduced HDL and ApoA1; Long-term SO_2_ associated with reduced HDL, ApoA1, and increased LDL, ApoB
Chen [98]	NHSII	25–42 years; 68%	107 532	20 years	PM_2.5_, PM_10_, PM_10-2.5_, NO_2_	HTN	none	Overall: Long-term PM_2.5_ associated with higher rate of HTN

*AHEI* alternative healthy eating index, *ApoA1* apolipoprotein A1, *ApoB* apolipoprotein B, *BKMR* Bayesian kernel machine regression, *BMI* body mass index, *CO* carbon monoxide, *HDL* high density lipoprotein cholesterol, *HMW* high molecular weight, *HTN* hypertension, *LDL* low density lipoprotein cholesterol, *Lp(a)* lipoprotein(a), *NHS* Nurses’ Health Study, *NHSII* Nurses’ Health Study II, *PM* particulate matter <2.5 (PM_2.5_), 2.5 to 10 (PM_10-2.5_), and <10 (PM_10_) μm in diameter, *NO*_2_ nitrogen dioxide, *O*_3_ ozone, *SES* socioeconomic status, *sOB-R* soluble leptin receptor, *SO*_2_ sulfur dioxide, *SWAN* Study of Women’s Health Across the Nation, *TG* triglycerides, *WC* waist circumference.

In the SWAN cohort, long-term NO_2_ and sulfur dioxide [96] and PM_2.5_ [97] were associated with adverse lipid profiles, and associations for PM_2.5_ were stronger in perimenopausal women compared to postmenopausal women [97]. Long-term PM_2.5_ was associated with higher rates of hypertension in NHSII [98] as well as in NHS, particularly among younger women (<65 years) [99]. Although not the focus of this review, several studies in SWAN identified associations between long-term exposure to air pollution and higher concentrations of CVD biomarkers (e.g., plasminogen activator inhibitor-1, C-reactive protein, and tissue-type plasminogen activator antigen) that varied by menopause status (e.g., [100, 101]). For example, particulate matter between 2.5 and 10 μm was associated with higher plasminogen activator inhibitor-1, especially in perimenopausal women [100].

## Discussion and future directions

Studies in midlife women suggest that exposure to nonpersistent and persistent chemicals, heavy metals, and air pollution increases risk for obesity and metabolic outcomes, and that the menopausal transition could represent a sensitive period of exposure. Additional research is needed to understand susceptibility to MDCs during perimenopause and identify modifiable factors to reduce exposure or mitigate adverse health outcomes. Below, we highlight research gaps and future directions on this topic.

### Sensitive periods of exposure

Although the perimenopausal period may represent a sensitive period of exposure, several analyses combined pre-, peri-, and postmenopausal women and not all studies examined differences in associations by menopause status, limiting the interpretation of findings for specific windows of exposure. Future studies during midlife may consider multiple time points of exposure assessment that span pre-, early peri-, late peri-, and menopausal periods coupled with statistical approaches for identifying susceptible periods (e.g., distributed lag models), which are commonly applied to environmental health studies in children [102]. Given the high variation in hormones between early and late perimenopause, as well as underlying changes occurring before the onset of perimenopause, capturing exposure during these distinct time windows is necessary for identifying sensitive periods for specific exposures.

### Life course approach

**Fig. 1:**
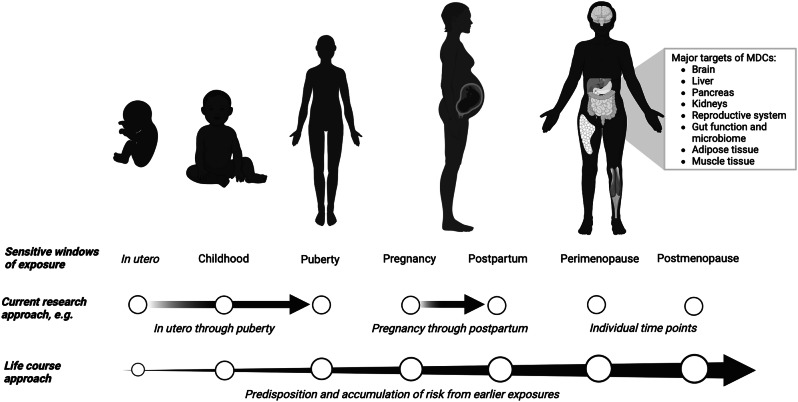
Life course approach: the menopausal transition as a sensitive window of exposure to metabolism-disrupting compounds. Exposure to MDCs across life stages, particularly during sensitive windows, can increase risk for adverse metabolic health by targeting several organs in the body. Current research focuses on individual time points or narrow life stages, such as exposure during fetal development through childhood and puberty or exposure during pregnancy on pregnancy-related outcomes or limited postpartum outcomes. In the life course approach, MDC exposure during sensitive windows may accumulate risk or predispose individuals to other metabolic risk factors. The menopausal transition, or perimenopause, represents a sensitive window for MDC exposure, and exposures during other sensitive windows over the life course could have cumulative roles in the development of metabolic outcomes and increase sensitivity to MDCs or other risk factors at later life stages. Abbreviations: MDC metabolism-disrupting chemicals or compounds. The figure was created with BioRender.com.

The life course approach is helpful for understanding the impact of MDCs on metabolic health [103]; exposure to MDCs during critical or sensitive windows may increase risk for metabolic outcomes later in life. MDC exposures during these windows may accumulate risk or predispose individuals to other metabolic risk factors (Fig. 1). Although this review focused on the menopausal transition, exposures during other sensitive windows over the life course, including fetal development, puberty, and pregnancy, could have cumulative roles in the development of metabolic outcomes and increase sensitivity to exposures at later life stages. For example, data from murine models suggest that prenatal phthalate exposure increases adiposity and glucose intolerance in adulthood, and underlying metabolic adaptations *in utero* in response to phthalate exposure increase susceptibility to a high-fat diet in adulthood [104, 105]. Future research efforts and funding mechanisms could leverage pregnancy cohorts for maternal follow-up throughout postpartum, perimenopausal, and postmenopausal periods to improve our understanding of long-term health consequences of MDC exposure across the lifespan.

### Multi-pollutant mixture methods

Most studies reviewed here focused on a single class of MDCs (e.g., phthalates, PFAS, heavy metals), and few studies used mixture methods to assess the cumulative impact of MDCs, which may have overlapping, synergistic, or antagonistic mechanisms of action. For example, exposure to PPARγ agonists can improve insulin sensitivity, which led to the development of pharmaceuticals such as thiazolidinediones to target PPARYγ ligands; however, several MDCs are linked to increased adipogenesis and adipocyte dysfunction by activating PPARYγ via ligand-dependent and -independent mechanisms, which could lead to intracellular insulin resistance [106]. Future studies should consider mixtures of several classes of MDCs and their cumulative associations with health outcomes, given likely overlapping mechanisms and non-monotonic relationships.

### Diverse populations

The majority of studies reviewed here were based in the U.S., with most data coming from the SWAN cohort. Although SWAN represents a diverse sample of women in the U.S., the subcohort of women with available chemical data excluded those of Hispanic ethnicity because Hispanic women were recruited from sites that did not collect biospecimens. The participants in MWHS were representative of the population in Baltimore, MD; given the small population of Hispanic women in this area, the Hispanic ethnicity is also underrepresented in this cohort. Other studies in midlife women included in this review (NHS, NHSII) were conducted within the U.S. and included participants who were majority White and college educated. Additional data in diverse populations both in the U.S. and other countries would capture a larger range of exposures and help identify particularly vulnerable populations.

### Susceptible populations

Several studies in this review stratified by biological factors such as BMI and social factors such as race/ethnicity to identify susceptible populations and better understand racial disparities in metabolic outcomes. The associations between MDCs and metabolic outcomes may differ by BMI due to underlying factors linked to adiposity, such as adipocyte function, hormone trajectories, or inflammation, that may interact with MDC mechanisms [47, 49]. Race/ethnicity may increase susceptibility to MDCs due to increased allostatic load driven by structural and interpersonal racism [107], while associations between MDCs with metabolic health may also be confounded or exacerbated by low-resource settings with limited food access [108]. Identifying susceptible populations during sensitive periods is important for risk assessment and informing policy [109].

### Accounting for lifestyle factors

Lifestyle factors such as diet are important sources of MDC exposure and may contribute to risk of developing adverse metabolic outcomes. Dietary intake of specific foods, such as fish as a source of PFAS [110], may underestimate the association between MDCs and adverse metabolic outcomes, whereas others, such as red meat as a source of phthalates [111], may lead to an overestimation of the relationship. Other dietary factors may modify the relationship between MDCs and health outcomes [112]. Several studies included in this review, but not all, accounted for intake of specific food items in main analyses [75, 83, 85–87] or sensitivity analyses [55, 58, 65, 67, 83] or accounted for overall diet quality [53, 59, 68], whereas others considered diet quality as a modifier [68, 93]. The consideration of diet as a confounder and/or modifier of MDC toxicity may improve our understanding of the role of MDCs in metabolic health.

### Mitigation strategies

Additional research is needed to understand strategies to mitigate adverse health outcomes during perimenopause. Dietary and lifestyle factors may reduce exposure to MDCs or target specific mechanisms, such as antioxidant intake to reduce oxidative stress [113]. Other studies in the SWAN cohort identified the pre- and/or perimenopausal period as a sensitive window for choline and betaine intake for improved cognitive outcomes [114], calcium supplementation for bone mineral density [115], and antioxidants for reduced fractures [116], suggesting opportunities for mitigating metabolic consequences of MDCs during this period. Interventions focused on diet as well as exercise and stress management have been shown to improve markers of metabolic health in individuals with metabolic syndrome [117, 118], and may represent potential strategies to improve health during perimenopause.

### Gut microbiome

MDCs are potential modulators of the gut microbiome—which functions as a metabolic organ [119]. The gut microbiome has a well-established role in the development of obesity and adverse metabolic health [120–124]. For example, evidence suggests fecal microbiota transplants from lean individuals reduce body weight in individuals with obesity [125]. Several reviews highlight the impact of MDCs—including heavy metals, phthalates, bisphenols, air pollution, and POP—on altered microbial metabolites, bacterial abundance and diversity, and intestinal permeability, with subsequent effects on body fat and glucose metabolism [126–130]. For example, fecal microbiota transplants from cadmium- or pesticide-exposed mice increased body fat in control mice [131, 132], and in humans, the associations of PFAS concentrations with insulin resistance and obesity were mediated by microbial-derived bile acids [133]. Conversely, the gut microbiome may influence the absorption, metabolism, and accumulation of MDCs, and their subsequent toxicity and body burden [134–137]. To our knowledge, no studies to date have investigated associations of MDCs with microbiome-related outcomes in perimenopausal women. Given that reproductive aging is also a potential modulator of the gut microbiome [38, 39], more data are needed on MDCs, gut microbiome, and metabolic health during perimenopause.

## Conclusion

Overall, studies suggest that phthalates and other nonpersistent chemicals, PFAS, POPs, heavy metals, and air pollutants were associated with increases in body fat, unfavorable adipokine profiles, adverse lipid profiles, and/or higher risk of type 2 diabetes and hypertension. Several studies identified differences by race/ethnicity. Although few studies stratified by menopause status, some results suggest that perimenopause may be a sensitive window of exposure. As research continues to focus on MDC exposure *in utero*, during childhood and puberty, and during pregnancy, additional research on MDCs is needed during the menopausal transition. Future research should consider examining cumulative exposure to multi-pollutant mixtures and identifying susceptible populations and mitigation strategies during this period. Given that the postmenopausal period represents up to 40% of a woman’s life, identifying modifiable factors during sensitive periods to mitigate MDC exposure could have important implications for long-term health.

## Supplementary information

SUPPLEMENTAL MATERIAL
