# Supplementary material for: Metabolism-disrupting compounds and metabolic health during the menopausal transition: a narrative review
Source: Int J Obes (Lond). 2026 Jun 15;50(7):1426–42. doi: 10.1038/s41366-026-02125-z (PMC13391365; doi:10.1038/s41366-026-02125-z)
Supplement: Supplementary file 1 — SUPPLEMENTAL MATERIAL [file 41366_2026_2125_MOESM1_ESM.docx]

**SUPPLEMENTAL INFORMATION**

| **Supplemental Table 1**. Search strategy for PubMed (conducted February 21, 2025) | | |
| --- | --- | --- |
| #1 | "Endocrine disruptors" [mesh] OR “Endocrine disruptor*” [tiab] OR “endocrine disrupting chemical*” [tiab] OR "persistent organic pollutant*" [tiab] OR dioxin* [tiab] OR dibenzodioxin* [tiab] OR ((perfluorinated [tiab] OR perfluoroalkyl [tiab] OR polyfluorinated [tiab] OR polyfluoroalkyl [ tiab]) AND (compound* [tiab] OR chemical* [tiab] OR substance* [tiab] OR acid*[tiab])) OR “polybrominated biphenyl*” [tiab] OR "Polybrominated diphenyl*" [tiab] OR “polychlorinated diphenyl*” [tiab] OR “polychlorinated dibenzofuran*” [tiab] OR “polychlorinated biphenyl*” [tiab] OR Bisphenol* [tiab] OR nonylphenol* [tiab] OR octylphenol* [tiab] OR dichlorophenol* [tiab] OR perchlorate* [tiab] OR "phthalic acid*"[tiab] OR phthalate* [tiab] OR paraben*[tiab] OR triclosan*[tiab] OR triclocarban* [tiab] OR oxybenzone*[tiab] OR benzophenone*[tiab] OR "Polycyclic aromatic hydrocarbon*" [tiab] OR pesticide* [tiab] OR insecticide* [tiab] OR herbicide* [tiab] OR fungicide* [tiab] OR atrazine [tiab] OR “2,4-dichlorophenoxyacetic acid”[tiab] OR organophosphate* [tiab] OR glyphosate* [tiab] OR Carbamate* [tiab] OR pyrethrin* [tiab] OR pyrethroid* [tiab] OR “organochlorine*” [tiab] OR "air pollution" [tiab] OR "air pollutant*" [tiab] OR "particulate matter" [tiab] OR PM2.5 [tiab] OR PM10 [tiab] OR “heavy metal*” [tiab] OR mercury [tiab] OR cadmium [tiab] OR arsenic [tiab] OR chromium [tiab] OR lead [mesh] | 582 518 |
| #2 | Menopause [mesh] OR menopaus* [tiab] OR premenopaus* [tiab] OR pre-menopaus* [tiab] OR postmenopaus* [tiab] OR post-menopaus* [tiab] OR perimenopaus* [tiab] OR peri-menopaus* [tiab] OR (midlife [tiab] AND women [tiab]) OR “menopausal transition” [tiab] | 136 947 |
| #3 | Metabolic syndrome [mesh] OR “Diabetes Mellitus, Type 2” [mesh] OR Adiposity [mesh] OR overweight [mesh] OR “waist circumference” [tiab] OR “body mass index” [tiab] OR “body fat” [tiab] OR “body weight” [tiab] OR metabolic [tiab] OR cardiometabolic [tiab] OR obesity [tiab] OR adiposity [tiab] OR diabetes [tiab] OR insulin [tiab] OR glucose [tiab] OR hypertension [tiab] OR “blood pressure” [tiab] OR adipokines [tiab] OR dyslipidemias [mesh] OR triglycerides [mesh] OR cholesterol [mesh] OR hyperlipemia [tiab] OR hyperlipidemia [tiab] OR dyslipidemia [tiab] OR dyslipidaemia [tiab] OR cholesterol [tiab] OR triglyceride* [tiab] OR “lipid profile” [tiab] | 3 204 310 |
| #4 | #1 AND #2 AND #3 | 297 |
